# Mapping the prevalence and nature of drug related problems among hospitalised children in the United Kingdom: a systematic review

**DOI:** 10.1186/s12887-019-1875-y

**Published:** 2019-12-11

**Authors:** Adam Sutherland, Denham L. Phipps, Stephen Tomlin, Darren M. Ashcroft

**Affiliations:** 1 Centre for Pharmacoepidemiology and Drug Safety, Division of Pharmacy and Optometry, School of Health Sciences, Manchester Academic Health Science Centre, University of Manchester, Manchester, M13 9PT UK; 2 Pharmacy Department, Royal Manchester Children’s Hospital, Manchester Universities NHS Foundation Trust, Oxford Road, Manchester, M13 9WL UK; 3 NIHR Greater Manchester Patient Safety Translational Research Centre, Manchester Academic Health Science Centre, University of Manchester, Manchester, M13 9PL UK; 4 Pharmacy Department, Great Ormond Street Hospital, Holborn, London, WC1N 3JH UK

**Keywords:** Drug related problems, Paediatric, Medication errors, Hospital in-patients

## Abstract

**Background:**

Problems arising from medicines usage are recognised as a key patient safety issue. Children are a particular concern, given that they are more likely than adults to experience medication-related harm. While previous reviews have provided an estimate of prevalence in this population, these predate recent developments in the delivery of paediatric care. Hence, there is a need for an updated, focussed and critical review of the prevalence and nature of drug-related problems in hospitalised children in the UK, in order to support the development and targeting of interventions to improve medication safety.

**Methods:**

Nine electronic databases (Medline, Embase, CINAHL, PsychInfo, IPA, Scopus, HMIC, BNI, The Cochrane library and clinical trial databases) were searched from January 1999 to April 2019. Studies were included if they were based in the UK, reported on the frequency of adverse drug reactions (ADRs), adverse drug events (ADEs) or medication errors (MEs) affecting hospitalised children. Quality appraisal of the studies was also conducted.

**Results:**

In all, 26 studies were included. There were no studies which specifically reported prevalence of adverse drug events. Two adverse drug reaction studies reported a median prevalence of 25.6% of patients (IQR 21.8–29.9); 79.2% of reactions warranted withdrawal of medication. Sixteen studies reported on prescribing errors (median prevalence 6.5%; IQR 4.7–13.3); of which, the median rate of dose prescribing errors was 11.1% (IQR 2.9–13). Ten studies reported on administration errors with a median prevalence of 16.3% (IQR 6.4–23). Administration technique errors represented 53% (IQR 52.7–67.4) of these errors. Errors detected during medicines reconciliation at hospital admission affected 43% of patients, 23% (Range 20.1–46) of prescribed medication; 70.3% (Range 50–78) were classified as potentially harmful. Medication errors detected during reconciliation on discharge from hospital affected 33% of patients and 19.7% of medicines, with 22% considered potentially harmful. No studies examined the prevalence of monitoring or dispensing errors.

**Conclusions:**

Children are commonly affected by drug-related problems throughout their hospital journey. Given the high prevalence and risk of patient harm,, there is a need for a deeper theoretical understanding of paediatric medication systems to enable more effective interventions to be developed to improve patient safety.

## Background

Preventable adverse events in healthcare account for substantial patient morbidity and mortality [1]. Approximately 850,000 patients in the National Health Service (NHS) in England experience an adverse event during their hospital stay, costing the NHS an additional £2bn per year [2]. Medication-related events account for 10–20% of all adverse healthcare events in the NHS, costing between £200–400 million per year [3]. A recent United Kingdom (UK)-focussed literature review of primary and secondary healthcare settings estimated that there were approximately 237 million medication errors every year in the NHS, 66 million of which are likely to be clinically significant and 29% were estimated to occur in secondary care settings [4]. Recently, the World Health Organisation (WHO) has made reducing medication related harm by 50% within 5 years its third global patient safety challenge [5].

### Definitions

**Fig. 1:**
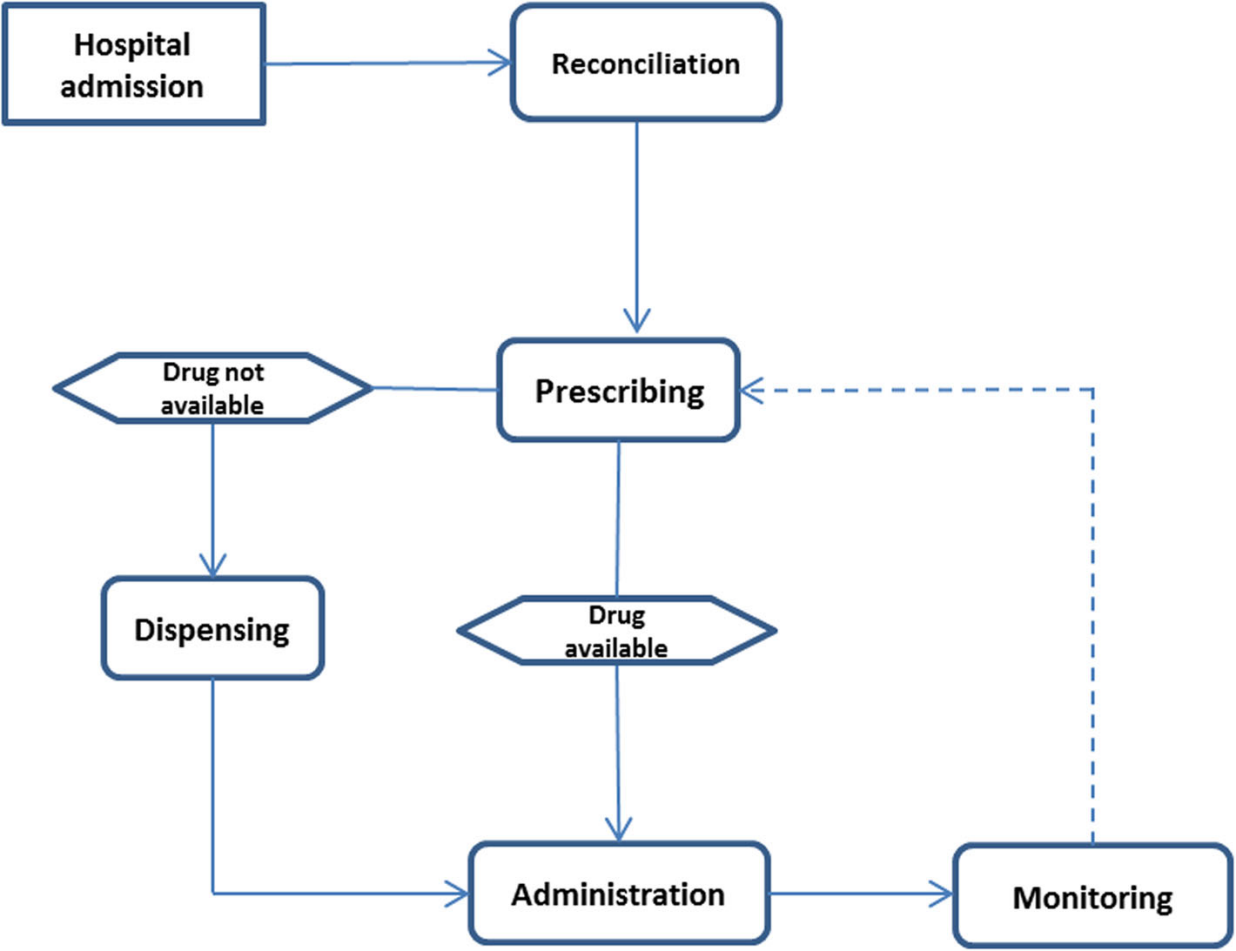
High-level process model of medication use in UK hospitals

The term “drug-related problems” (DRPs) has been used to encompass a range of potential or actual negative health outcomes as a result of medication use [6]. Operationally, DRPs are a composite classification of events including safety (expressed as medication errors (MEs)), effectiveness (defined as adverse drug reactions (ADRs)) and necessity (described as unnecessary drug use.) Outcomes of DRPs are operationalised as adverse drug events (ADEs). However, the way DRPs are defined varies across the literature, with DRPs as a single concept for evaluating medication use having as many as twenty different systems for categorisation [7]. There has been considerable interest in safety- and effectiveness-related DRPs in the UK. ADEs, ADRs, and MEs are related (Fig. 1), with preventability being the key differentiator between events [8]. ADEs are events related to medication that result in an adverse outcome, and many are preventable [9]. An ADR is “…a response to medicine which is noxious and unintended, and which occurs at doses normally used in man.” [10]. An ADE occurs when the drug is administered in a manner or at a dose not usually used in humans (resulting for example from a wrong-route administration, or administration of an over- or under-dose). However, many published studies have used these terms interchangeably, which can complicate the direct comparison of ADR studies.

An ME has been defined by the National Co-ordinating Committee’s Medication Error Reduction Programme (NCC-MERP):

*“A medication error is any preventable event that may cause or lead to inappropriate medication use or patient harm while the medication is in the control of the health care professional, patient, or consumer. Such events may be related to professional practice, health care products, procedures, and systems, including prescribing, order communication, product labeling, packaging, and nomenclature, compounding, dispensing, distribution, administration, education, monitoring, and use.”* [11]

However, Ghaleb identified 26 discrete operational definitions for MEs which also make direct comparison of studies difficult [12].

Children are estimated to be three times more likely than adults to experience harm related to their medication [9]. The reasons for this are uncertain. It may be related to an increased complexity in the approach to medication used for children [13]. Paediatric medicines optimisation requires an individualised approach to drug therapy, sometimes in the absence of robust trial data or suitable medication formulations, thus introducing greater potential for harm [14]. Miller identified that errors in paediatric medication occurred at all stages of the medicines process – prescribing (3–37%), dispensing (5–58%), administration (72–75%) and documentation (17–21%) [15]. While Miller’s was a global review, it covered only 5 years from 2000 to 2005. A 2006 systematic review of the frequency and nature of paediatric medication errors identified that dosing errors (prescribing and administration) and drug selection errors were the most common [12]. A 2001 systematic review of adverse drug reactions (ADRs) in hospitalised children estimated a global incidence of 9.5% with severe reactions accounting for 12.3% of these reactions [16].

Understanding the epidemiology of drug-related problems is important to inform efforts by healthcare professionals, researchers, patients and carers to mitigate these problems through improvement interventions. However, the reviews cited are now over 10 years old, and predate the publication of a number of major strategy papers and interventions to improve paediatric medication safety [13, 17, 18]. Also, it has been suggested that because of methodological inconsistency, comparison and extrapolation of event rates across geographical and regulatory boundaries may not be appropriate [19].

For these reasons, this paper reports an updated systematic review in this area to include more recent studies on drug-related problems in hospitalised children, and provides a more granular assessment of prevalence and nature of drug-related problems in the UK.

## Methods

**Fig. 2:**
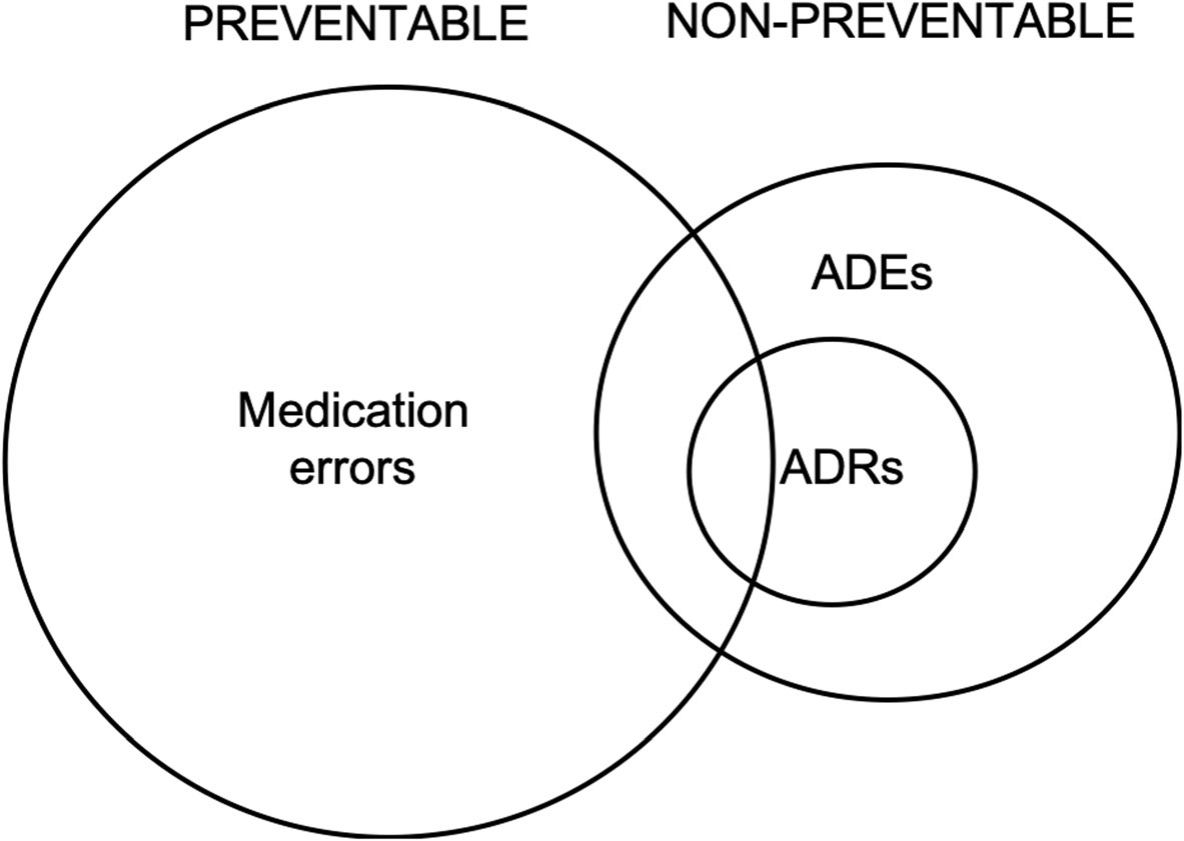
The relationship between MEs, ADEs and ADRs adapted from Bates et al. [8]

We undertook a systematic review of the literature relating to the prevalence and nature of drug-related problems in hospitalised children in the United Kingdom. The review was conducted in line with the Preferred Reporting Items for Systematic Reviews and Meta-analyses (PRISMA) guidelines [20] and the review protocol was registered with the International Prospective Register of Systematic Reviews (PROSPERO; 118,535). For the purposes of this review, “Drug Related Problems” refers to indicators of safety - ADEs, ADRs and MEs. The definition used and operationalised in each study was extracted and compared. To contextualise the review, we adapted a conceptual map of the medication process in hospitalised children from the model proposed by Walsh et al. [21] Walsh’s model describes a process whereby prescribers documented medication orders into the medical note, and nurses transcribed these into an administration record, reflecting the medication system in the United States at the time; however, in the UK medication orders are predominantly prescribed onto charts that serve as both prescription and administration record [22]. In recent years there has been a concerted effort to move to computerised physician order entry (CPOE) but the extent of this is uncertain [23]. UK hospital wards also hold a range of medication as stock therefore dispensing is often captured as part of the administration process for stock drugs [24]. A separate “dispensing” step was included for non-stock items obtained from a pharmacy. Additionally, medicines reconciliation has become an important part of the medication system in UK hospitals to ensure continuity of care [25] and this important step was added to our process model as “reconciliation”. Thus, the medication process model presented in Fig. 2 was used.

### Search strategy

Nine electronic databases (Medline, Embase, CINAHL, PsychInfo, International Pharmaceutical Abstracts, Scopus, Health Management Information Consortium, the British Nursing Index, The Cochrane library and International clinical trial databases) were searched from January 1st 1999 to September 18th 2018. Searches were re-run in July 2019 to include the period from September 2018 to April 30th 2019. 1999 was selected as this marked the publication of “To Err is Human” [26], which stimulated considerable research on the subject of medication-related harm. The grey literature including publicly available government reports, were also searched through the OpenGrey portal (www.opengrey.eu) for studies that met the inclusion criteria. The reference lists of all included studies were hand searched to identify any additional citations. The search strategy was constructed with reference to search terms derived from previously published systematic reviews [12, 27, 28], and with the support of a university librarian. The strategy is summarised in Additional file 1: Table S1.

### Inclusion and exclusion criteria

The review included studies published in English, presenting data relating to the prevalence of DRPs in hospitalised children and young people in the United Kingdom. For the purposes of this review, children were defined as anyone under the age of 18 years of age [29]. Studies that reported data on MEs, ADRs and ADEs were included. Eligible study designs included observational epidemiological studies (including cross-sectional and cohort studies) and interventional studies (randomised controlled trials, non-randomised controlled trials, before-and-after studies and interrupted time series) where pre-intervention data was reported. Conference abstracts were included where they provided sufficient data to enable the calculation of an event rate for the outcome of interest, with a clear denominator expressed.

Systematic and narrative reviews were excluded, but their reference lists searched for eligible studies. To ensure inclusion of all eligible studies the authors of suitable studies that collected paediatric data but did not present these separately (e.g. studies reporting all population groups) were contacted for access to data sets to permit extraction by the review team. Studies based on spontaneous incident reporting data were also excluded as denominators in these studies are often imprecise and the prevalence not determined. Furthermore, studies on adherence to medication were excluded as hospital in-patient medication is administered by nursing staff and adherence could reasonably be assumed to be captured as “omitted doses.”

A post-hoc amendment to the protocol was made after abstract screening, when it was found that a large proportion of potentially eligible studies (*n* = 7) were rejected purely due to their being set in Paediatric and Neonatal Intensive Care Units. In the original protocol these studies were to be excluded because of the differences in admission patterns and physiological dynamics of these patients. Given the number of such studies, the review team agreed that critical care should be included.

### Data extraction and synthesis

Studies were screened against the inclusion criteria initially by title, and subsequently by abstract. Full text articles were further screened against inclusion criteria by the lead author (AS). Included articles were then reviewed in duplicate, independently by members of the review team (AS, DLP, DMA) and data extracted using a purpose developed and piloted proforma. This proforma collected descriptive details of each study – year of publication, country (England, Wales, Scotland or Northern Ireland), the clinical setting, study design and the duration. Other information extracted included the definitions used and method of data collection, their outcome of interest (ADEs, MEs, or ADRs) including the denominator, and the stage of the medication process at which these events occurred – admission and discharge (intended to include issues arising at transition of care), prescribing, dispensing, administration and monitoring.

**Fig. 3:**
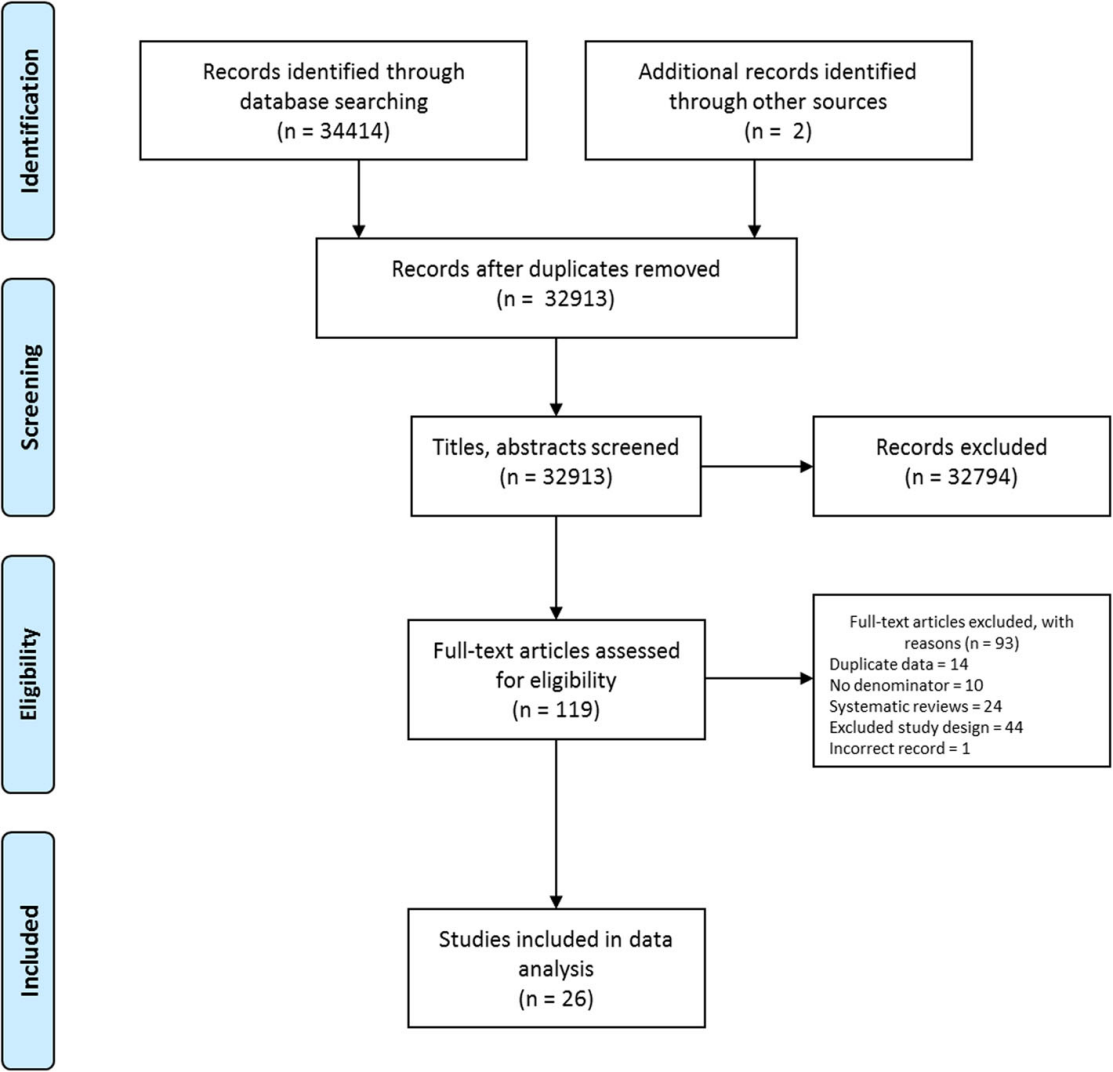
Flow chart of study assessment for eligibility

The primary outcome of interest was the prevalence rate of MEs, ADRs and ADEs identified at each stage of the medication process. Data on the severity and preventability of events were also extracted. Data were summarised descriptively in tables, and prevalence rates were summarised at each stage of the medication process. All studies were observational cross-sectional in nature, with marked differences in the way outcomes were operationalised between studies. This heterogeneity amongst the studies made meta-analysis inappropriate; however, within studies of similar design and denominator, results were summarised at each stage as median rates to provide an estimate of the prevalence overall, and interquartile ranges were calculated to provide a measure of the variation in outcomes between studies. Study quality was assessed using Allan and Barker’s method for medication error studies, adapted by Ghaleb and Wong [12, 30]. 11 criteria were reviewed for each study (Fig. 3).

Box 1 Study quality criteria

**Table:** 

• Clearly stated aims and objectives • Clearly stated phenomenon of study – ME/ADE/ADR • Categories of ME/ADE/ADR specified and defined • Clearly stated definition of phenomenon of study • Clearly described method of detection • Clearly stated setting • Clearly stated denominator (or ability to calculate one from the data) • Clearly described sample size and sampling method • Description of reliability measures • Description of validity measures • Listing of limitations • Description of assumptions made	

## Results

In total, 26 studies were identified that met the inclusion criteria. A summary of the flow of studies through the screening and assessment process is shown in Fig. 3, and descriptive information of the included studies is presented in Additional file 2: Table S2.

### Study settings, design and process stages

Twenty-three studies were set in English NHS contexts (two multi-centre, 6 in the North of England, 3 in the Midlands and 12 in London) with two in Scotland and one in Northern Ireland. One study examined both prescribing and administration [31]. Seven studies were set in or included critical care data – six in Paediatric Intensive Care (PICU) [31–36] and one in Neonatal Intensive Care (NICU) [37]. There were three studies examining medicines reconciliation (1 on discharge and 2 on admission to hospital) [38–40], 16 studies explored MPEs [31–37, 41–49]; six studies examined medication administration (four studying MAEs [31, 50–52], and two studying the incidence of adverse drug reactions.) [53, 54] Two studies examined the incidence of DRPs as a specific concept using the Pharmaceutical Care Network Europe (PCNE)classifications [55, 56]. There were no studies relating specifically to ADEs, or to DRPs associated with dispensing of medication or monitoring of therapy.

Three studies used a retrospective approach [41, 45, 46] and 23 used prospective designs. Retrospective studies all used longitudinal cohort study designs ranging from 1 month to 1 year, while prospective studies lasted between 1 day and 10 months. Prospective studies used cross-sectional observational designs, with two studies using an interrupted time series design [44, 49].

### Definitions of drug related problems

All three medicines reconciliation studies used consistent methodology and definitions, with discrepancies between the best possible medication record and the documented medication history as the unit of analysis. For the purposes of this review, these discrepancies were treated separately from prescribing errors, as they have been studied and defined differently. All studies of DRPs in prescribing related to the study of medication prescribing errors (MPEs.) Three prospective studies used a similar definition related to clinical significance derived from Ghaleb [57], which enabled comparison [31, 47, 48]. Seven studies MPEs as deviation from local and national policy therefore presented a different assessment of potential errors or deviations from recommended practice [34, 37, 42–44, 49]. Two studies reported data on technical prescribing errors, defined as a failure to complete prescriptions in line with local policy [44, 47]. Five studies did not define “error” [32, 33, 36, 41, 45, 46].

Two studies of medication administration errors used the same definition, which was based on independent assessment of the likelihood of errors to cause harm to the patient [31, 50]. A third prospective study of medication administration errors used an outcomes-based definition to separate “errors” (drug-related problems that may lead to actual harm to the patient) from “discrepancies” (deviations from policy or procedure that would not lead to harm to the patient) [51]. One study used quantitative accuracy as a surrogate of medication administration problems (measurable doses) but made no link to patient outcomes [52].

ADR studies used two different (but similar) definitions. Bellis and Thiesen used the definition of ADR from Edwards and Aronson [58], and Rashed used the World Health Organisation definition [10]. Both definitions purport to exclude medication errors.

### Study quality

A summary of the study quality is presented in Additional file 3: Table S3. Thirteen studies met 10 to 12 of Allan & Barker’s criteria and were evaluated as Category A (Results should be accepted as reported) studies, with clear reporting of definitions, validity and reliability methods. Of these studies seven were multicentre, therefore generalisability of the remaining 6 studies is questionable. Eight studies stated statistical power for detection of the event being studied. All observational studies used site-based data collectors, however only five studies described how these data collectors were trained [31, 38, 50, 51, 55]. Ten studies used subject matter experts or independent review of classification to enhance the reliability of the events recorded [38–40, 44, 47, 48, 51, 53, 54, 59].

### Assessment of severity

Seven studies evaluated the potential harm associated with DRPs; two were related to ADRs [53, 54] and three to discrepancies detected during medicines reconciliation [38–40]. One study examined the potential harm associated with prescribing errors [48] and one reported potential harm of administration errors [51].

The ADR studies used two differing methods for evaluating potential harm. Rashed [53] used the Dormann method for evaluating the severity of ADRs [60] and identified that 136 (61%) ADRs were rated as “mild,” 85 (38.1%) “moderate” and 2 (0.9%) “severe.” Conversely, Thiesen [54] used the Hartwig scale [61] and identified that 322 (22.1%) ADRs were level 1 (no harm), 1112 (76.9%) were level 2 or 3 (drug held but no lasting harm) and 13 (1%) associated with harm (12 level 4 and 1 level 5.) No fatal or otherwise prolonged harm events were identified.

All three medicines reconciliation (MR) studies used the consensus method described by Cornish to evaluate potential severity of medication discrepancies [62]. For the MR on admission studies, 50–78% of discrepancies were rated to be moderate or severely harmful, while on discharge 22% were rated moderately harmful with no severely harmful discrepancies.

One prescribing error study [48] utilised the consensus method described by Dean et al. to evaluate potential harm [63]. More than half of these errors (63.5%) were considered to be moderate or severely harmful. A prospective observational study on intravenous (IV) medication administration errors used an outcome-related harm categorisation (NCC-MERP [64]) to differentiate errors from discrepancies [51]. None of the MAEs noted in this study were associated with any harm.

**Fig. 4:**
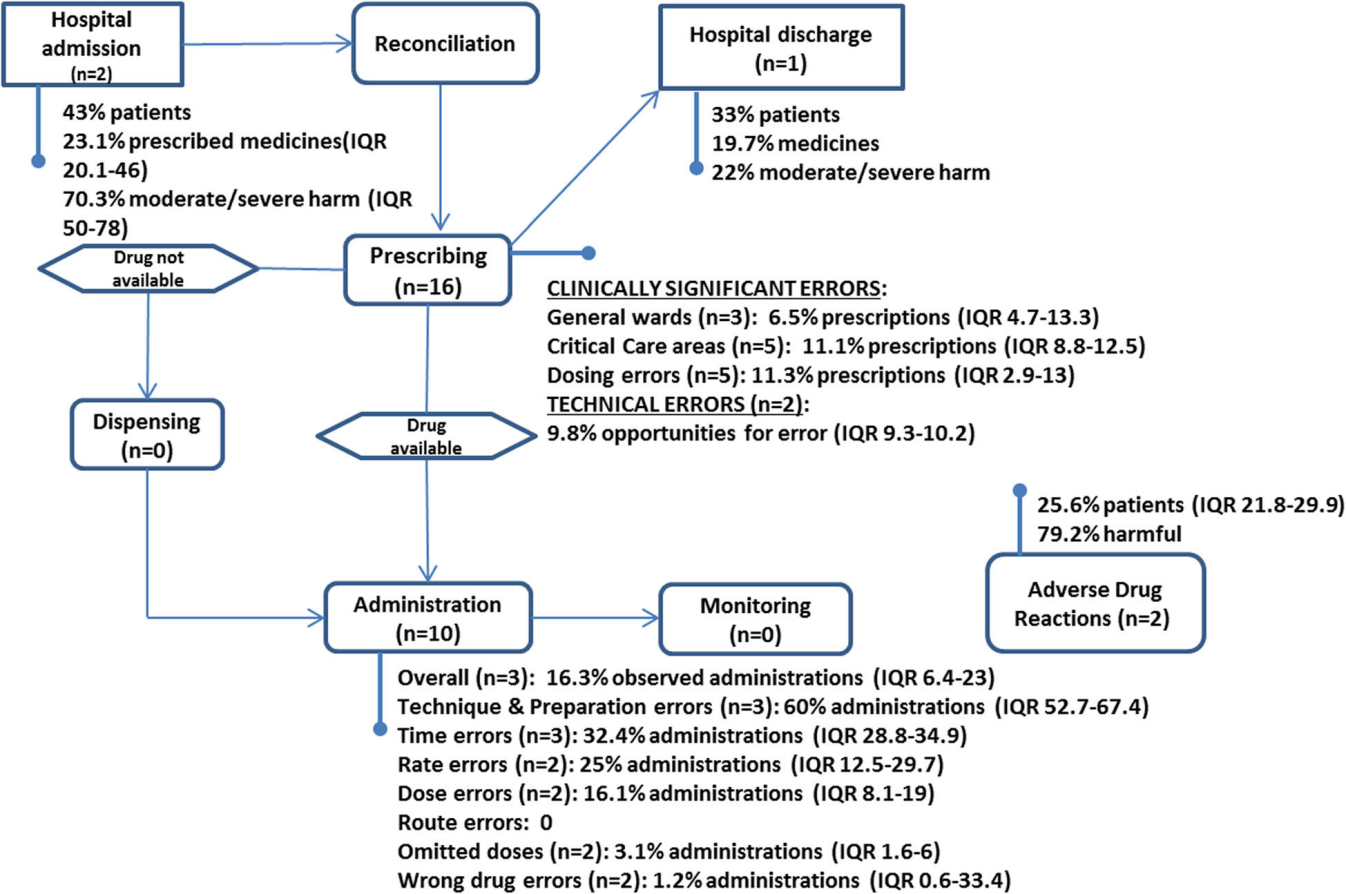
Process map and prevalence of DRPs

The distribution of DRPs in the medication process is presented in Fig. 4.

### Medicines reconciliation

The results of the three MR studies are compared in Table 1.

**Table 1:** Results from medicines reconciliation studies

Study	Admission						
	Pts	Drugs	Discrepancies (%)		Potential severity (%)		
			Patients	Drugs	Mild	Moderate	Severe
Huynh [38]	244	1004	109 (45%)	209 (20.8%)	22 (22%)	50 (50%)	28 (28%)
Terry [40]	39	97	Not stated	45 (46%)	19 (50%)	11 (29%)	8 (21%)
Discharge							
Huynh [39]	142	501	47 (33%)	99 (19.7%)	77 (78%)	22 (22%)	Nil

At admission to hospital the median rate of medication discrepancies was 23.1% of documented orders (Range:20.1–46). Only Huynh cited the number of patients affected (45%). 70.3% (Range 50–78) of discrepancies on admission were deemed clinically significant (those rated as moderate or severe.) whereas only 22% of discrepancies on discharge met this threshold.

### Medication prescribing

Sixteen studies included data on MPEs. Seven studies [31, 42–44, 47, 48, 65] used the intensive chart review method described by Ghaleb (using a clinician, pharmacist or pharmacy staff member as primary data collector) as their data collection method [31]. However, only three of these studies [31, 47, 48] collected data on clinically significant errors based on similar definitions [57] and these have been summarised (Table 2). One study [31] also included PICU and NICU data which was extracted and presented separately. Across the three studies, the median prevalence of MPEs was 6.5% (IQR 4.7–13.3).

**Table 2:** Prevalence of MPEs in Paediatric Wards

	Orders	Errors	%
Ghaleb [31]	2955	391	13.2
O’Meara [48]	1911	125	6.5
Lepee [47]	657	31	4.7

It was possible to extract data on error rates for dosing errors from five studies [31, 41, 42, 47, 48]. These data are presented in Table 3. The median rate of dosing errors was 11.2% of medication orders (IQR 2.9–13).

**Table 3:** Prevalence of dosing errors . (P) – prospective; (R) – retrospective. PICU/NICU data has been excluded

	Observed orders	Dosing	
		Errors	%
Bolt (R) [41]	99	13	13
Davey (R) [42]	76	46	60.5
Ghaleb (P) [31]	391	44	11.2
Lepee (P) [47]	657	17	2.6
O’Meara (P) [48]	1911	56	2.9

### Critical care settings

Seven studies in critical care settings utilized prospective chart review methods to explore the prevalence of MPEs. The duration of studies was variable, and ranged from 96 h [36] to 36 weeks [35] with two studies not stating a duration [32, 34]. Six studies used the number of prescriptions observed as their denominator, and the results are summarised in Table 4. The median prevalence of MPEs in critical care was 11.1% of medication orders (IQR 8.8–12.5).

**Table 4:** Prevalence of MPEs in Paediatric and Neonatal Critical Care Settings

	Orders	Errors	Prevalence of MPEs (%)
Sutherland [32]	815	94	11.5
Warrick [36]	159	14	8.8
Morris [33]	376	47	12.5
Isaac [34]	1896	909	47.9
Fordham [37]	292	16	5.5
Ghaleb [31]	1020	109	10.7

One study [35] used occupied bed days (OBD) in a single Paediatric Intensive Care Unit (PICU) as the denominator and identified a rate of MPEs of 892 per 1000 OBD.

The definitions of MPE differed across these studies, with three studies not stating a definition [32, 33, 36]. Two studies used local definitions [34, 37], one study used Ghaleb’s definition [31], and one study used the definition of MPE proposed by Bates [35].

Two studies reported the nature of MPEs. Isaac found that 82% of prescribing errors were “technical” in nature (incomplete prescriptions) affecting 39.2% of prescriptions [34]. The prevalence of clinically significant errors was 13.9% of prescriptions. Booth also found that technical errors were more prevalent than clinical errors (394/1000 OBDs and 230/1000 OBDs respectively.) [35] Additionally, this study identified that prescriptions for continuous intravenous infusions accounted for 30.4% (295/892) of MPEs identified with incorrect or omitted infusion rates being most common (31.2 95%CI 26.2–36.7).

### Technical prescribing errors

Two studies used opportunities for error to explore technical MPEs which allowed comparison [44, 47]. The median prevalence was 9.8% (IQR 9.3–10.2). One study [42] used the number of prescriptions as the denominator (76/249, 30.5%) and one study [43] expressed a rate per-10-drug charts as the denominator (32/12 averaged to 27/10). Woodley used a retrospective study design and identified a prevalence of MPEs of 90.9% of drug prescriptions, compared against in-house standards [49]. None of these studies explored the potential severity of the errors.

Three studies did not state standards or definitions for MPEs [41, 45, 46]. Bolt and Lane used retrospective chart reviews to assess MPEs in paediatric dental services and cleft services respectively. MPE rates were observed between 13 and 100% of prescriptions with dosing errors being most common, however only Bolt provided a standard against which dosing was compared.

### Medication administration

Three studies used direct observation to identify MAEs in paediatric clinical areas [31, 50, 51]. .Two studies were multicentre – one studying MAEs in paediatrics and the other studying intravenous MAEs in adult and paediatric practice [31, 51]. One single-centre study observed nurse double checking of medication [50]. The authors of one study were contacted for paediatric data as this was not reported separately [51]. As described earlier, there were differences in the definitions of “error” used across the studies. Alsulami included “parental administration of medication without nursing observation” as a unique error type which has been suggested to inflate error estimates [66]; therefore these errors (64/191) were excluded from our analysis.

These studies are summarised in Table 5 and the median rate of MAEs was 16.3% of opportunities for error (IQR 6.4–23).

**Table 5:** Frequency and nature of MAEs. Columns and rows will not sum to the error value as it is possible for one administration to represent more than one error. Alsulami’s error type “administered by parent” removed from analysis

	Observed administration	Errors (Prevalence)	Tech/Prep (%)	Wrong time (%)	Wrong rate (%)	Wrong dose (%)	Omit dose (%)	Wrong drug (%)
Alsulami [50]	2000	127(6.4%)	95 (74.8)	32 (25.2)				
Ghaleb [31]	1074	247(23%)	112 (45.3)	80 (32.4)	85 (34.4)	40 (16.2)	22 (8.9)	3 (1.2)
Lyons [51]	196	32(16.3)		12 (37.5)	8 (25)	7 (21.8)	1 (3.1)	21 (65.6)
Total	3270	406	207 (51%)	124 (30.5)	93 (22.9)	47 (11.6)	23 (5.7)	24 (5.9)
Median (IQR)		16.3% (6.4–23)	60%(52.7–67.4)	32.4% (28.8–34.9)	25% (12.5–29.7)	16.1% (8.1–19)	3.1%(1.6–6)	1.2%(0.6–33.4)

One study evaluated dosing accuracy as a surrogate of potential MAEs. Morecroft [52] retrospectively studied 1599 prescribed doses of intravenous and oral medication for 431 patients in three paediatric wards over 5 weeks and observed 196 unmeasurable doses (12.3%). “Measurability” was defined based on the availability of enteral syringes and assumptions about the strength of liquid medications that were available on the wards. Doses of less than 5 ml accounted for 75.5% of these unmeasurable doses.

### Adverse drug reactions

Two studies evaluated the incidence and nature of ADRs in paediatric practice using prospective observational study designs and are summarised in Table 6.

**Table 6:** Prevalence of ADRs

	Patients	Pts with > 1 ADRs	%
Thiesen [54]	5118	906	17.7
Rashed [53]	297	101	34.0

The median rate of ADRs was 25.9%. The differences in prevalence can be explained methodologically, with Rashed including possible and unlikely ADRs which were excluded from Thiesen’s analysis. Furthermore, Thiesen included only patients admitted > 48 h in the study sites, whereas Rashed enrolled at 24 h, thus there is uncertainty in both estimates. Across both studies, severity of the ADRs was assessed and 79.2% of reactions were severe enough to warrant discontinuation of therapy.

### Drug related problems as a specific outcome

Two studies were identified focussing on drug related problems (DRPs) as a specific outcome [55, 56]. Both studies used different versions of the Pharmaceutical Care Network Europe classification system of DRPs (including markers of safety, necessity and effectiveness) [67]. Neither of these studies could be included in the comparisons as the PCNE system is not intended for outcome-related assessment, but to explore contributing and causative factors. However, both studies serve to provide an interesting insight into the complexities of medication use in children and young people in hospital, and therefore merit inclusion. Ibrahim studied the prevalence and nature of DRPs in renal patients in two tertiary paediatric centres in London [55]. 127 patients were screened and 203 DRPs across 166 charts were identified, with a prevalence of 51.2% (95%CI 43.2–60.6%). The most common DRPs were sub-optimal drug effect (44/203, 21.7%) and unnecessary treatment (41/203, 20.2%). Rashed et al. explored the prevalence of DRPs in a single children’s hospital in London over 3 months as part of an international multi-centre study [56]. 373 patients were reviewed and 147 patients with DRPs were identified (39.4, 95%CI 34.4–44.6%). The most common DRPs were problems with drug selection (43/93, 46.2%) and problems with drug dosing (41/93, 44.1%).

## Discussion

This systematic review has identified that in the UK, DRPs remain common throughout the in-patient medication process for hospitalised children from admission to discharge. Adverse drug reactions affect more than one in four hospitalised children. Documentation errors on admission affect 43% of patients, with 70% of these errors likely to cause harm. One in fifteen children is affected by a clinically significant prescribing error, and this prevalence increases to one in 10 children in critical care units. Half of all prescribing errors were a result of incorrect dose selection. Technical prescribing errors occurred almost twice as often as clinical errors. However, our findings suggest that MAEs are the most common ME, affecting one in six medications administered. Two thirds of these errors are related to preparation of medicines or administration technique, with practitioners unable to administer medicines at the right time in one third of administrations, or administer a quarter of IV medications at the right rate.

The estimates in this study must, however, be considered alongside the limitations of the included studies. Very few studies used comparable denominators, operational definitions and data collection methods; this points to the need for the greater standardisation in future studies.

The results of this review offer some interesting contrasts with a similar recent review. Gates et al. attempted to meta-analyse ME estimates across the global literature [68], and encountered the same definitional and methodological challenges encountered in this review. MPEs were more prevalent in critical care areas (25.9%; 95%CI 17.3–36.7) compared to general ward areas (14.7% (95%CI 6.1–31.6). However, technical and clinically significant errors were grouped together in a number of studies, which may explain our lower estimates as we have endeavoured to separate these where possible. Conversely, the MAE estimates in Gates’s review are far lower than ours (3.1% of observed administrations (0.4–19.5) in multiple wards). While this may reflect differences in inclusion criteria (the large scale multi-centre study of IV medication errors [51] was excluded as children did not account for > 90% of the sample.) this cannot solely explain our estimate. Gates argues that lower-quality studies resulted in higher estimates, and thus the MAE estimate is taken from “high” quality studies. However the range of MAEs in Gates’s study using similar denominators to included MAE studies in our review is 0.2–89.9 MAEs per 100 administrations. Thus we can offer a more granular, regional estimate of the prevalence of paediatric MAEs in the UK.

This review has identified few studies reporting ADEs. It may be argued that the ADR studies offer some insights into the extent of ADEs. Across all ADR studies, a change to medication was required in more than 70% of identified cases, and Rashed identified that 12.6% of ADRs were “serious” (resulted in hospital admission, lasting harm or death.) 17.9% of observed ADRs in Thiesen’s cohort were considered to be preventable. Of the studies included in this review, those that reported on DRP severity found that most were low/no harm. This is likely to be an unrealistic estimate. Panagioti and colleagues have found that preventable medical harm occurred in 6% of patients, and 12% of these caused severe harm or death [1]. 25% of these events were caused by medication. However, paediatric estimates are lacking. Panagioti’s finding is consistent with existing estimates of the prevalence of preventable ADEs in hospitalised children. In New Zealand this was 1.3% [69] in a 12-week study in a general paediatric unit. Similarly in the USA this was 1.2% [70]. There have been no studies of ADEs in the United Kingdom thus the scope of potential and preventable harm is unknown.

This review provides insights into the wider systemic problems with medication safety in the UK. The prevalence of ADRs in the UK identified in an international study is almost five-fold higher than comparable health systems (Germany and Australia) identified in the same study [53]. Further this estimate is double that reported in another single-centre prevalence study (34 and 17.7% respectively) [54]. Both studies used the same detection method, but chose different rating systems for potential ADRs. Thiesen used a panel of subject matter experts who agreed on causality of all potential ADRs but included only probable or definite ADRs. Rashed evaluated only twenty ADRs with an independent review panel with only fair correlation (k = 0.3). This study also included “possible” ADRs (24.7% of observed ADRs) in the analysis. Thus Rashed may represent an overestimate that can be explained methodologically.

Notwithstanding these methodological limitations, the findings suggest that there remains considerable scope for improvement in medicines optimisation for hospitalised children in the UK. Our review has highlighted that ADR prevalence in hospitalised children is substantially higher than that reported in UK adults.(14.7%) [71] The use of opiates is higher in the UK than in other care contexts and are associated with a high incidence of ADRs. Further, off-label and unlicensed medicines are implicated in a large proportion of paediatric ADRs and this continues to be a problem in children and young people [72].

MAEs are the most frequent DRP in UK children in hospital, with rate, dose and preparation errors being most prevalent among them. These findings complement a recent global systematic review of MAEs, who found that wrong time errors were most common in paediatrics, followed by preparation and dosage errors, with administration technique and rate the third most prevalent error subtype [73]. This reflects the lack of standardised methods for preparation and administration of medicines especially in children, where adaptation of adult formulations is often necessary for administration [74].

There are also significant problems around documentation of medication information, particularly with regards to medication histories and documenting prescriptions. As discussed earlier, the UK continues to have a largely paper-based prescribing system, which presents issues around accuracy and completeness of prescriptions. This is reflected in the large number of MPE studies identified in this review. Ghaleb et al. estimated the prevalence of MPEs in the UK to be 13.2% of prescriptions. However, in comparison with other similar studies, we suggest that the prevalence of MPEs is lower, and closer to prevalence rates in adult populations. The pivotal EQUIP study including 20 hospitals in the North of England identified a rate of MPEs of 8.8% of prescriptions (95%CI 8.6–9.1) [75]. The estimate in our review (6.5% of prescriptions; IQR 4.3) is based on comparison of three studies that used similar definitions and denominators, however it must be noted that Ghaleb reported a higher incidence than the other studies, but this study included technical errors in prescribing (failure to complete prescription documentation) which may account for the higher estimate.

We have identified that dosing errors are the most common MPE in hospitalised children and young people. This has been known for some time [76] but appears to remain a problem. There is likely to be a strong human component in the aetiology of these errors. Jani et al. [65] studied the impact of electronic prescribing in a British children’s hospital, and reported an overall reduction in dosing error rates from 2.2 to 1.2% (95%CI -1.6 – − 0.5). However in this study, the in-patient rate of dosing errors did not change (1.42 to 1.39%, *p* = 0.95). Further study of this system identified a high rate of rejection of duplicate-dosing alerts that was later found to be caused by the design of the electronic prescribing system whereby legitimate and appropriate prescriptions were triggering alerts [77]. This also supports the earlier work of Potts et al. [78] who found that while technical prescribing errors (properly formatted and completed prescriptions) were reduced by 99.4%, potential ADEs were reduced by 40.9% with dosing errors unaffected. In electronic prescribing systems, it has been found that poor design and implementation can lead to worsening of safety, and even increased mortality [79]. Thus there is a suggestion of cognitive deficit in the choice and calculation of doses that has not yet been identified.

Amongst MPEs, technical errors – the failure to complete a prescription in full – were the most prevalent. This is a theme that was identified through the PICU studies with infusion documentation particularly prone to error [34, 35]. This may be a result of the predominance of bespoke, weight based infusions that are commonplace in PICU [80]. However complex, multi-calculation prescribing requirements for these infusions are associated with errors and inaccuracy and ADEs [81–83]. This complexity of prescribing may also be connected with MAEs. Included in this review, Morecroft’s MAE study identified that 12.3% of prescribed doses were unmeasurable using available medicines [52]. This is supported by evidence from operating theatres and pharmacy aseptic units, where dosing accuracy is directly affected by the volume of concentrated drug required to deliver the dose. Where the dose is < 1 ml in volume, inaccuracy in the measured dose increases exponentially, independently of the operator or their experience level [82, 84]. Several qualitative studies have explored the underlying factors associated with MPEs and MAEs in hospitals [74, 85–87]. Together these studies suggest that the causes of errors are very much dependent on the environmental, personal and organisational context. These are important considerations n the development of medication safety interventions.

Only a limited number of studies examined the severity of DRPs, however two studies reported ADRs which were common. No information on ADEs was found. In the studies that did consider severity, most of the DRPs in this review are of low severity or no harm. Thus it would appear that there are systems in place that ultimately mitigate the harm of errors at the bedside. Recent literature has highlighted the importance of technological interventions (barcoding medication, “Smart” pumps, electronic prescribing and automated drug dispensing) and unit-based pharmacists to improve paediatric medication safety [88]. However, there is little or no understanding about how effective these interventions are in the UK context.

This review included a number of interventional studies which can shed light on existing attempts to improve medication safety in UK paediatric units. In a behavioural interventional study, Booth et al. studied a “zero tolerance” approach to prescribing errors in PICU that consisted of dedicated prescribing areas, strict enforcement of prescribing standards and tailored feedback [35]. This study found a reduction in all-cause MPEs of 44.5% (95%CI 40.8–48%) Other interventional studies in this review have focussed on educational initiatives (feedback, didactic teaching, simulation and assessment) [37, 42–44, 49]. All were uncontrolled single centre studies, but reported reductions in DRPs.

Pharmacists were primary data collectors in these studies demonstrating their important contribution to detection and resolution of DRPs and yet there are no paediatric interventional studies evaluating the impact of a ward-based pharmacist in the UK. In international studies, pharmacists detect and resolve a large number of MEs, with > 95% acceptance of these interventions [70, 89, 90]. However it may be difficult to undertake controlled studies of the contribution of pharmacists to medication safety in hospitalised children and young people as pharmacists are now embedded in UK practice. There is increasing evidence of the qualitative benefits of pharmacists in paediatric care. An ethnographic study identified that pharmacists were key to medication-related decision making in specialist paediatric hospitals in Australia [91]. Further, a UK qualitative study on prescribing errors in PICU found that pharmacists were an important control for medication error, but were only present on an ad hoc basis [87].

An aspect of this review that merits further study is the complexity of medication systems in hospitals. A system is defined as “…a set of elements or parts that is coherently organised and interconnected in pattern or structure that produces a characteristic set of behaviours…” [92] It has been suggested that hospital environments are complex socio-technical systems where humans are expected to interact with increasingly complex systems in order to deliver care [93–96]. With the drive to improve safety in the NHS by introducing more technology, there is a need to understand these systems and how people and technology work together to ensure patient safety.

In considering future research priorities, there is a need for a systems-based understanding of how medication systems function in NHS hospitals in order to theoretically inform the design of interventions to improve patient safety. Additionally, there is a clear need for future work evaluating the burden of harm caused to hospitalised children and young people as a result of preventable ADEs.

## Conclusions

Our findings show that children are affected by DRPs throughout their journey in hospital. While prescribing errors appear to be no more prevalent than in adults, the increased prevalence of ADRs suggests that there is much potential medication related harm in hospitalised children and young people. However, the incidence of preventable ADEs is uncertain as all UK medication studies to date have been process orientated with no outcome focussed research identified, thus there is an urgent need for outcome-focussed research on preventable ADEs in paediatric hospital settings in the UK.

A deeper understanding of medication processes for children in hospital from a systems and theoretical perspective will also support the development and targetting of effective interventions to improve patient safety.

## Supplementary information

**Additional file 1.** Search strategy.

**Additional file 2.** Study Summary.

**Additional file 3.** Summary of study quality.

## Data Availability

The datasets analysed in this manuscript are available from the corresponding author on reasonable request.

## Abbreviations

ADE: Adverse Drug Event
ADR: Adverse Drug Reaction
CDSS: Clinical Decision Support System
CPOE: Computerised Physician Order Entry
DRP: Drug Related Problem
MAE: Medication Administration Error
ME: Medication Error
MPE: Medication Prescribing Error
NHS: National Health Service
PICU: Paediatric Intensive Care Unit
PRISMA: Preferred Reporting Items for Systematic reviews and Meta-Analysis
PROSPERO: International Prospective Register of Systematic Reviews
UK: United Kingdom
WHO: World Health Organisation
