# Supplementary material for: Mapping the prevalence and nature of drug related problems among hospitalised children in the United Kingdom: a systematic review
Source: BMC Pediatr. 2019 Dec 11;19:486. doi: 10.1186/s12887-019-1875-y (PMC6905106; doi:10.1186/s12887-019-1875-y)
Supplement: Supplementary file 1 — Additional file 1. Search strategy. [file 12887_2019_1875_MOESM1_ESM.docx]

| **Concept** | **Search term** | **Limiters** |
| --- | --- | --- |
| Concept 1 - Population | P*ediatric* (as key word) | TI, AB |
|  | Neonate (Pre term) |  |
|  | Neonate (term) |  |
|  | Infant |  |
|  | Child |  |
|  | Adolescent |  |
| Concept 2a - Setting | In-patients |  |
|  | Ward |  |
|  | Unit |  |
|  | Ambulatory Care Unit |  |
|  | Hospital discharge |  |
|  | Hospital admission |  |
| Concept 3 – Drug-related problems | Drug Related Problem* (as key word) |  |
|  | Adverse Drug Event |  |
|  | Adverse Drug Reaction  OR Drug Reaction  OR Medication reaction |  |
|  | Medication error  OR Drug error  OR Prescribing error  OR Administration error  OR Dispensing error  OR Monitoring error |  |
|  | Omitted doses  OR missed doses  OR delayed doses |  |
|  | Medicines reconciliation  OR medication history  OR drug history |  |
